# Advances in Cell Engineering of the *Komagataella phaffii* Platform for Recombinant Protein Production

**DOI:** 10.3390/metabo12040346

**Published:** 2022-04-14

**Authors:** Cristina Bustos, Johan Quezada, Rhonda Veas, Claudia Altamirano, Stephanie Braun-Galleani, Patrick Fickers, Julio Berrios

**Affiliations:** 1 School of Biochemical Engineering, Pontificia Universidad Católica de Valparaíso, Av. Brasil 2085, Valparaíso 2362803, Chile; cristinabustos_vero@hotmail.es (C.B.); j.quezadaolguin@gmail.com (J.Q.); rhonda.veas@pucv.cl (R.V.); claudia.altamirano@pucv.cl (C.A.); stephanie.braun@pucv.cl (S.B.-G.); 2 Microbial Processes and Interactions, TERRA Teaching and Research Centre, Gembloux Agro-Bio Tech, University of Liège, Av. de la Faculté 2B, 5030 Gembloux, Belgium; pfickers@uliege.be

**Keywords:** *Komagataella phaffii*, *Pichia pastoris*, recombinant protein, cell engineering, metabolic engineering

## Abstract

*Komagataella phaffii* (formerly known as *Pichia pastoris*) has become an increasingly important microorganism for recombinant protein production. This yeast species has gained high interest in an industrial setting for the production of a wide range of proteins, including enzymes and biopharmaceuticals. During the last decades, relevant bioprocess progress has been achieved in order to increase recombinant protein productivity and to reduce production costs. More recently, the improvement of cell features and performance has also been considered for this aim, and promising strategies with a direct and substantial impact on protein productivity have been reported. In this review, cell engineering approaches including metabolic engineering and energy supply, transcription factor modulation, and manipulation of routes involved in folding and secretion of recombinant protein are discussed. A lack of studies performed at the higher-scale bioreactor involving optimisation of cultivation parameters is also evidenced, which highlights new research aims to be considered.

## 1. Introduction

The methylotrophic yeast *Komagataella phaffii,* formerly known as *Pichia pastoris* [1], is one of the most prominent recombinant protein (rProt) production platforms [2,3]. As a methylotrophic yeast, it can oxidise methanol for energy production and biomass formation [4]. Given its ability to grow readily on relatively inexpensive culture media, it was initially used as a cellular protein source (SCP, single-cell protein). Over the years, it has become an attractive host system for the production of rProt from bacterial, fungal, plant, and mammalian/human origins [2,5,6,7].

*K. phaffii* stands out among other yeasts and microorganisms due to several beneficial features (for more information, see an excellent comprehensive review from Ata et al. [8]). In addition to its ability to metabolise methanol through the methanol utilisation (MUT) pathway, *K. phaffii* can grow on inexpensive media at high cell density, reaching a biomass concentration exceeding 100 g/L of dry cell weight (DCW) [9,10,11]. Genetic manipulation tools for transgene expression in this species are well-established, resulting in the targeted and stable integration of rProt genes. Proteins carrying a suitable secretion signal, such as the widely utilised alpha mating factor (MATα) secretion signal, can be accumulated in the culture supernatant due to its efficient secretory machinery in an environment relatively free from other proteins and contaminants, as less than 10% of the endogenous proteins are secreted [2,12]. In addition, *K. phaffii* can perform diverse protein processing and post-translational modifications typical of higher eukaryotes, such as glycosylation and disulphide bond formation [3,13], and it lacks other known disadvantages that are present in bacterial systems (formation of inclusion bodies and presence of endotoxins) or mammalian cell systems (high cultivation and handling costs) [14]. Using this host system, rProt can be produced in either a constitutive or induced manner, depending on the type of promoter used to drive recombinant gene expression.

The success of *K. phaffii* for rProt synthesis has been facilitated by strong methanol-inducible promoters from the alcohol oxidase genes (alcohol oxidase 1, *AOX1*, and to a lesser extent, alcohol oxidase 2, *AOX2*), and also from the glyceraldehyde 3-phosphate dehydrogenase (GAP) promoter *P_GAP_,* which exhibits strong constitutive expression in the presence of glucose and glycerol [15]. Notwithstanding the efficiency, tight control and rProt productivity obtained when using *P_AOX1_* to drive transgene expression, this production system presents some drawbacks associated with methanol utilisation. Indeed, methanol is toxic to cells, inducing cell oxidative stress, and its use comes with a subsequent high oxygen demand for catabolism [16]. Additionally, as methanol is highly flammable, its use can imply safety issues, especially at an industrial scale. Taking these drawbacks into consideration, current research is being undertaken to evaluate alternatives in order to reduce or discard methanol use.

**Figure 1:**
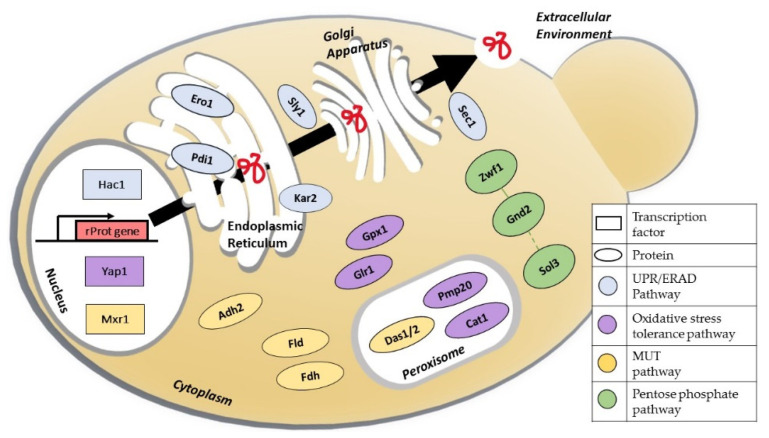
Schematic overview of the main pathways, proteins, and transcription factors involved in cellular engineering strategies for the improvement of recombinant protein production in *Komagataella phaffii*. Abbreviations: Hac1: UPR-regulating transcription factor; Yap1: oxidative stress response transcription factor; Mxr1: methanol expression regulator 1; Pdi1: protein disulphide isomerase; Kar2: immunoglobulin-binding protein; Ero1: endoplasmic reticulum oxidoreductase; Sly1: hydrophilic protein involved in ER/Golgi vesicles trafficking; Sec1: Sm-like protein involved in docking and fusion of exocytic vesicles; Gpx1: glutathione peroxidase; Glr1: glutathione reductase; Pmp20: peroxisome-membrane-associated protein 20; Cat1: catalase; Das1/2: dihydroxyacetone synthase 1 and 2; Fld: formaldehyde dehydrogenase; Fdh: formate dehydrogenase; Zwf1: glucose-6-phosphate dehydrogenase; Sol3: 6-gluconolactonase; GND2: 6-phosphogluconate dehydrogenase.

Important efforts have been carried out in order to improve the understanding of the physiology and cell response of *K. phaffii* under various genetic backgrounds (engineered strains) and bioprocess operations [17,18,19] with the goal of increasing rProt productivity, cell capabilities and fitness, and metabolic performance. These advancements have been reported in several reviews that focus on bioreactor processes [20,21], genetic manipulation techniques [22,23,24], and metabolic engineering [25]. However, there is a need to highlight and summarise the latest cell engineering approaches and strategies regarding manipulation of the methanol pathway, co-factor metabolism, transcription modulation, protein folding and secretion, as well as catabolism of alternative carbon sources, as illustrated in Figure 1, and their contribution to *K. phaffii*’s promising future as a robust and highly efficient host for the production of a huge variety of recombinant proteins.

## 2. Metabolic Engineering for Improved Metabolism and Energy Supply

rProt production and secretion generate a high metabolic burden, as protein synthesis leads to increased nutrient and energy demands [26]. In addition, production of rProt can often trigger endoplasmic reticulum (ER) stress and oxidative stress, causing (NAD(P)H) co-factor unbalance [27]. As stated above, *K. phaffii* can metabolise methanol as the sole carbon source; however, it can also metabolise other non-frequently used alkylated nitrogen sources, such as methylamine and choline. The main metabolic drawback of methanol catabolism is the production of toxic metabolites such as formaldehyde and hydrogen peroxide [28]. At high methanol concentration (above 5% vol/vol), disruption of the peroxisome can occur, thus impairing methanol catabolism [29,30]. Furthermore, methanol catabolism requires high oxygen consumption that can limit the productivity of the bioreactor process, especially at large scale where the oxygen transfer capacity is lower [31]. Therefore, the methanol concentration control in the culture medium is crucial to obtain high productivity of rProt.

In processes based on the *P_AOX1_* expression system, co-substrates such as sorbitol can be used with the goal of dedicating methanol mainly as the inducer for the expression system, while sorbitol is used for biomass and energy formation [32,33,34]. This has been evidenced by metabolic flux analysis of a simplified metabolic network describing cell growth, methanol and sorbitol catabolism, and energy formation, which was subsequently confirmed in a bioreactor culture [35]. In this case, it was observed that an appropriate methanol/sorbitol mixture ratio (methanol fraction 0.60 C-mol/C-mol) could increase the *P_AOX1_* induction level (β-galactosidase activity of 8.6 ± 0.8 × 10^3^ Miller unit), compared to cultures with 100% methanol supplementation (7.8 ± 0.7 × 10^3^ Miller unit) [35]. Glycerol has also been widely used as a co-substrate, and different culture strategies have been implemented. Recently, a combined μ-stat (constant exponential feeding rate) and m-stat (constant methanol concentration) feeding process was developed for β-glucosidase FBG1 production in a 5 L bioreactor. This co-stat feeding strategy allowed reaching a productivity of 403 mg/L of β-glucosidase, which was 2.6- and 4.4-fold higher than the titre obtained in μ-stat and m-stat modes, respectively [36].

Metabolic engineering of the host cell has also been considered, aiming at improving rProt productivity. Recent published work on engineering of the catabolic pathway of methanol and alternative carbon sources, along with co-factor engineering, is described in the following sections and summarised in Table 1.

### 2.1. Engineering of the Methanol Catabolic Pathway

**Figure 2:**
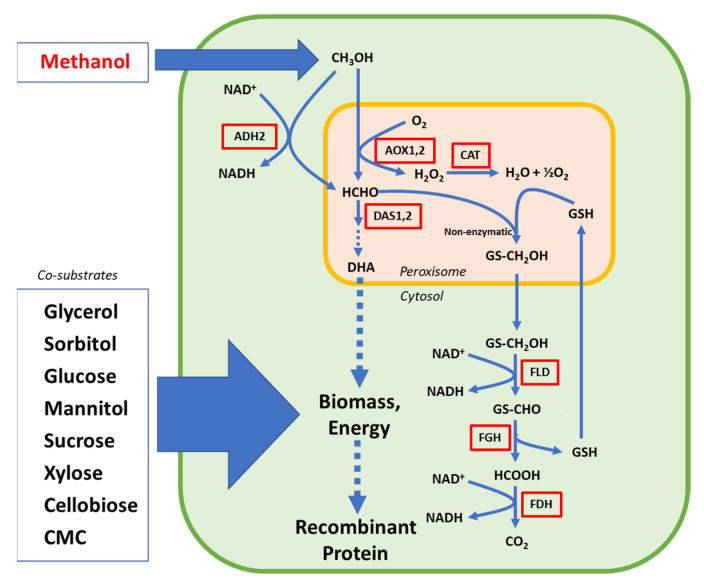
Diagram illustrating the main steps in the MUT pathway and co-substrate alternatives for enhancement of biomass and energy supply in *Komagataella phaffii.* AOX: alcohol oxidase; CAT: catalase; DAS: dihydroxyacetone synthase; FLD: formaldehyde dehydrogenase; FGH: S-formylglutathione hydrolase; FDH; formate dehydrogenase; ADH: alcohol dehydrogenase; GS(H): glutathione; CMC: carboxymethyl cellulose.

The MUT pathway can be divided into two main stages. First, methanol is oxidised by two alcohol oxidases (Aox1, Aox2) into formaldehyde in the peroxisome, before being further metabolised in two distinct metabolic routes. In the assimilatory branch, formaldehyde is converted into dihydroxyacetone (DHA) and GAP by the peroxisomal dihydroxyacetone synthase (Das). This pathway ends in the cytosol with the generation of fructose 1,6-biphosphate. In the dissimilatory branch, formaldehyde is oxidised to carbon dioxide in three steps by the enzymatic route formed by formaldehyde dehydrogenase (Fld), formyl glutathione hydrolase (Fgh), and formate dehydrogenase (Fdh), with the release of NADH [45], as illustrated in Figure 2.

Engineering of the MUT pathway has led to higher substrate-product conversion yield (Y_S/P_) and rProt productivity. In a Mut^S^ strain (strain with disrupted *AOX1*), the overexpression of *DAS* resulted in a 2- to 3-fold increase in Y_S/P_ from methanol to produce horseradish peroxidase (Hrp) and *Candida antarctica* lipase B (CalB), with reported values of 3.06 U/mmol and 2.05 U/mmol, respectively, in comparison with the reference strains for each case (~1 U/mmol). Meanwhile, the overexpression of *FLD* with the same reporter proteins exhibited a 2-fold increase in Y_S/P_ from methanol (1.65 U_HRP_/mmol and 2.13 U_CalB_/mmol) [37]. On the other hand, the deletion of both *DAS* isoforms (*DAS1* and *DAS2*) has increased green fluorescent protein (GFP) expression in single and double knockout strains. The highest rProt production was observed in the *DAS1* deletion strain, which produced 30% more GFP than the control strain. This yield was followed by the *DAS2* deletion strain and finally the double mutant *DAS1*-*DAS2*, with GFP expression levels of ~22% and ~15% in each case, both higher than the wild-type strain [38].

An alternative methanol metabolism implemented for recombinant protein production has been presented by Zavec et al. [46] with the recent reassessment of the Mut- strain (strain with *AOX1* and *AOX2* disruption), which, contrary to its generally recognised inability for methanol metabolization [47], showed that it is indeed able to metabolise methanol with a similar rProt yield when compared to a Mut^S^ strain, due to the promiscuous activity of the alcohol dehydrogenase enzyme Adh2. Based on these results and using metabolic engineering, the overexpression of *ADH2* revealed a significant increase in the productivity (q_P_) of the camelid antibody fragment vHH (237 μg/g∙h) in the Mut^-^*P_AOX1_*vHHpFLD1Adh2 strain compared to the Mut^-^*P_AOX1_*vHH parent strain (88 μg/g∙h), and a slightly higher increase with the strain Mut^S^*P_AOX1_*vHH (205 μg/g∙h). It is worth noting that despite the similarity in productivity between the Mut^-^ improved strain and the Mut^S^ strain, the latter showed an additional advantage by having lower rates of oxygen uptake and heat production [39].

As far as the main mechanism for induction of the *P_AOX1/2_* promoters is concerned, few studies have ventured to question whether methanol could be the sole inducer of the system. Tyurin and Kozlov [40] discussed the exclusive inducing activity of methanol and proposed the use of formate as a possible inducer of the *P_AOX1/2_* system. Thus, to rule out the possibility of intracellular methanol reduction using potassium formate salt as an inducer, *FDH* was deleted. With this modification, the expression levels of β-galactosidase were increased by 2-fold (~4500 Miller units) compared to the wild-type strain (~2200 Miller units) [40].

Modifying the MUT pathway and the redistribution of metabolic flux for rProt expression in key metabolic steps directed to a target product has also been successful [37,38,39,40]. However, little attention has been paid in recent years to modifying this pathway to improve the production of different recombinant proteins than those abovementioned, and some high-value compounds, such as malic acid and S-adenosylmethionine, have been produced when a knockout of *FDL* and *DAS* was introduced [48,49]. Thus, the work presented by Zavec et al. [39] and Tyurin and Kozlov [40] highlights the need to question the previously acknowledged and accepted premise of the MUT pathway behaviour and re-evaluate methanol metabolism from different perspectives.

### 2.2. Engineering of Co-Substrate Catabolic Pathways

Due to the already described problems with the use of methanol in rProt production, co-feeding strategies of methanol with auxiliary carbon sources have been studied to mitigate this problem. Based on the results of Inan and Meagher [50], it was identified that substrates such as sorbitol and mannitol could be used as co-substrates in combination with methanol, as these do not repress the *P_AOX1_* promoter, while glycerol and glucose do exert repression of this inducible promoter. Nevertheless, glycerol is mainly used in co-feeding strategies with methanol because it generates higher biomass yield, higher growth rate, and improved productivity [33]. The use of glucose has been favoured due to the deletion of the hexose transporter Hxt1, which made it possible to obtain a strain for the expression of rProt in a methanol-free medium [51]. On the other hand, the co-feeding with sorbitol positively affects cell growth and energy supply for heterologous protein production, which makes it one of the most widely used co-substrates to produce rProt in methanol-inducing media [31,33,52]. In order to improve xylanase expression under sorbitol-methanol co-feeding conditions, the gene identified in *K. phaffii* as the initial acceptor of the pexophagy process (*ATG30*) was deleted to favour peroxisome retention in cells under the influence of the addition of carbon sources that trigger this cellular response. This modification improved xylanase production, reporting an activity of ~1140.7 U/mL in cultures supplemented with 2% sorbitol, representing an increase of ~11.4% compared to the control culture containing 0.5% sorbitol [41]. This demonstrates that by employing cell engineering strategies, the production of a highly in-demand enzyme in the food and paper industry can be successfully increased [53].

*K. phaffii* does not normally metabolise sucrose because of the absence of an invertase enzyme [54]. However, a study evaluating the influence of the carbon source on cell size and production of an anti-LDL-single-chain variable fragment (scFV) [55], expressed in *K. phaffii* the Suc2 invertase enzyme from *Saccharomyces cerevisiae* [56] under the control of *P_AOX1_*. When this strain was grown in sucrose-supplemented medium, both the antibody fragment concentration (93.7 mg/L) and the specific yield on biomass (3.96 mg/g DCW) showed comparable values to glycerol-supplemented cultures (72.7 mg/L and 3.04 mg/g DCW, respectively). Additionally, when evaluating the use of glucose, this condition showed the lowest productivity (63.3 mg/L), even though using this medium generated a higher cell volume compared to sucrose or glycerol (0.766 μm^3^ in glucose, 0.214 μm^3^ in sucrose, 0.202 μm^3^ in glycerol) [55,57]. This suggests that high production of this heterologous protein in *K. phaffii* in sucrose-based cultures may be mainly related to cell number rather than cell concentration, in addition to the repressive effect on the *P_AOX1_* promoter from both glycerol and glucose [55,57].

The study of these auxiliary carbon sources in recent years has mainly focused on managing the conditions and operating parameters in culture systems [20,28,35,58]; therefore, an interesting and complementary scope of study could be carried out looking into the modification in their respective metabolic pathways, in conjunction with the manipulation and optimisation of these cultivation parameters.

### 2.3. Engineering of Turnover Co-Factor Metabolism

The metabolic burden that arises from processing rProt folding, posttranslational modifications, and secretion has shown that the regeneration of the oxidised co-factor significantly affects energy metabolism, becoming a bottleneck [42,59,60]. The overexpression of genes encoding enzymes catalysing NADPH-producing reactions has proven its usefulness for overcoming redox unbalance. An example of this is the overexpression of the *S. cerevisiae POS5*-encoded NADH kinase, which increased 2-fold the specific productivity of a Fab antibody fragment [27].

Constitutive overexpression of the genes encoding the glucose-6-phosphate dehydrogenase (*ZWF1*) and 6-gluconolactonase (*SOL3*) that catalyse NADPH-generating reactions led to a 3.8-fold increase in the productivity of human superoxide dismutase (hSod) [42]. Besides this, overexpression of *SOL3* and the gene encoding 6-phosphogluconate dehydrogenase (*GND2*) resulted in a 2.2-fold increase (~6.5 mg/L) in recombinant human interferon gamma (hIFN-γ) in comparison with the control strain GS115/hIFN-γ (2.5 mg/L) [43].

### 2.4. Engineering for Alternative Carbon Source Catabolism

In the context of circular economy, the trend has been the valorisation or increased added value of renewable raw materials, such as agricultural, forestry, food and industry bioproducts and/or waste streams. This goal would require *K. phaffii* to incorporate additional metabolic skills in order to metabolise substrates such as xylose, cellobiose, and cellulose [44,61].

The heterologous expression of genes involved in xylose catabolism, namely the xylose isomerase (*XI*) from *Orpinomyces* spp. and an endogenous xylulokinase, allowed xylose conversion resulting in a yield (Y x/s) of 0.378 g/g, nearly 2-fold higher than that of the parental strain GS115, reaching values comparable to those achieved with glucose or glycerol (0.310 g/g and 0.435~0.490 g/g, respectively) [44]. Using the same engineered strain, the β-mannanase production titre reached approximately 80 U/mL.

In a different research study, the expression of three heterologous cellulases, namely *Aspergillus niger* β-glucosidase (AnBGL1), *A. niger* endoglucanase (AnEG-A), and a *Trichoderma reesei* exoglucanase (TrCBH2), allowed the resulting strain to grow on cellobiose and carboxymethyl cellulose [61].

Another metabolic engineering strategy in the search for alternative carbon source metabolism consisted of increasing the acetate tolerance in *K. phaffii*. Indeed, acetate, which can be obtained from syngas by the hydrolysis of cellulosic biomass or by anaerobic fermentation of different agroindustrial wastes [62], is a cheap and largely available carbon source. This increased tolerance was conferred by the *P_GAP_*-driven overexpression of the native gene *PAS_Chr3_1091* encoding the putative serine/threonine protein kinase Hrk1, together with the *S. cerevisiae* gene ScAcs1* encoding acetyl-CoA [63].

Recently, *K. phaffii* was engineered for CO metabolization through a reorganisation of the MUT pathway as well as the xylulose monophosphate (XuMP) pathway, in order to generate a Calvin–Benson–Bassham resembling cycle. This modification was obtained by the expression of eight recombinant proteins from five different organisms: from *Ogataea polymorpha* (glyceraldehyde-3-phosphate dehydrogenase Tdh3, phosphoglycerate kinase Pgk1), *Ogataea parapolymorpha* (transketolase Tkl1, triosephosphate isomerase Tpi1), *Spinacia oleracea* (phosphoribulokinase Prk), *Thiobacillus denitrificans* (ribulose-1,5-bisphosphate carboxylase-oxygenase RuBisCO), and *Escherichia coli* (chaperones GroEL and GroES); and the deletion of three native genes (*AOX1*, *DAS1*, *DAS2*) [64]. This strategy opens up new opportunities for rProt synthesis from alternative carbon sources.

## 3. Transcription Factor Engineering in *K. phaffii* for Recombinant Protein Production

Numerous investigations have focused on how to modify gene expression patterns and regulatory networks during rProt production [65,66,67]. The growing understanding of cellular transcriptome modulation during highly stressful processes, such as rProt synthesis in high-density cultures [68,69,70], has allowed identifying regulatory elements of interest for the improvement of production processes [71,72,73]. These regulatory elements may regulate not only the promoter used for the recombinant gene expression, but also key steps of rProt synthesis, such as posttranslational modifications and secretion [74]. Recent research on this regard is summarised in Table 2.

For instance, Yap1 has been identified as a transcription factor associated with the control of the oxidative stress response by modulating the expression level of more than 150 genes [87,88]. Overexpression of the Yap1 encoding gene led to a 2-fold increase in the expression of the recombinant gene encoding trypsin, reaching at least 80 µg/g WCW (wet cell weight) of trypsin under the regulation of *P_GAP_* [79].

Several groups have also investigated transcription factors associated with protein maturation and secretion. The protein Hac1 is a transcription regulator involved in the unfolded protein response (UPR), which increases the synthesis of endoplasmic reticulum resident proteins required for protein folding, as well as components of the secretory pathway [74,82]. Overexpression of this transcription factor has proven to be a very important tool to increase the production yield of different recombinant proteins [81,85,86,89,90,91,92,93,94]. Huang et al. [93] evaluated the effect of HAC1p on a raw starch hydrolysing α-amylase (Gs4j-amyA) to improve heterologous production of the enzyme in *K. phaffii*, further evaluating the variation in copy number and promoter used in the overexpression of *HAC1* [93]. In this case, a strain with 12 copies of the *GS4J-AMYA* gene driven by *P_AOX1_* and a basal expression of 305 U/mL was used, and upon incorporation of six copies of *P_AOX1_*-driven *HAC1*, amylase activity increased to 2200 U/mL. However, the excessive number of genes under the control of the *AOX1* promoter seemed to interfere, limiting the transcription of *GS4J-AMYA*; therefore, by using the *P_GAP_* constitutive promoter to regulate the expression of *HAC1*, the amylase activity increased to 3700 U/mL. This illustrates the importance of the strategy followed with each auxiliary gene (in this case *HAC1*), this being as important as the choice of the auxiliary gene itself.

In some cases, an improvement can be achieved by decreasing or eliminating the expression of an auxiliary gene. One example of this comes from the transcriptional factor associated with galactose metabolism *ATT1*, a homologue of the *GAL4* gene in *S. cerevisiae*, which specifically recognises sequences called galactose upstream activating sequence (GALUAS) and is mainly associated with genes related to sugar metabolism [71]. In relation to this, glycol-engineered *K. phaffii* strains are very valuable tools to produce more complex recombinant glycoproteins [95]. However, in addition to enabling the production of rProt with human-like glycosylation patterns, glycoengineered strains also change the glycan structures of all endogenous glycoproteins [78]. Although the exact physiological consequences of such widespread glycan remodelling are not well-understood, it is evident that modifying the glycosylation pathway can affect the overall fitness of the host cell [96,97]. In this context, Jiang and colleagues reported that silencing *ATT1* increased the rProt yield with a 1.5-fold change in comparison to the control strain, reaching 1.98 g/L of human epidermal growth factor receptor 2 (anti-HER2) mAb. In addition, this increased the cell temperature tolerance by enduring 35 °C for up to 150 h with a low cell lysis rate in a 15 L bioreactor [78].

Additionally, Ata et al. [98] constructed a synthetic library based on *P_GAP_*, where they found 41 putative transcription factor binding sites (TFBS) in *K. phaffii*. As *P_GAP_* is a carbon-source-related promoter [15], TFs related to carbon source utilisation and their corresponding binding sites were considered as potential targets. Based on this, 10 strain variants were generated with deletion or duplication of TFBS, and the yield and effect of each modification was evaluated with the intracellular production of eGFP and/or extracellular production of recombinant human growth hormone (rhGH) as reporter proteins. The best results were obtained with the duplication of TFBS associated with the overexpression of the Gal4-like transcription factor; combining these two approaches resulted in a 2.2-fold increase in specific rhGH yield compared to *P_GAP_* in fed-batch bioreactor cultivation [98]. The specific glucose consumption and ethanol production rates were increased by 1.7- and 8-fold, respectively, in the *K. phaffii* Gal4-like transcription factor overexpressing mutants when compared to the wild-type, and it was noticed that the overexpression of the Gal4-like transcription factor resulted in a switch from Crabtree negative to Crabtree positive behaviour [71].

A similar approach was carried out for *P_AOX1_* to convert it into a methanol-free expression system [99]. The individual overexpression of two out of the three known transcription factor genes involved in regulation of the MUT pathway, namely *MRX1* and *MIT1*, activated *P_AOX1_* when the glycerol as repressing carbon source was depleted (derepressed expression) [77]. In parallel, Wang et al. [100] reported that the knockout of the three transcription factors *MIG1*, *MIG2*, and *NRG1* alongside *MIT1* overexpression also resulted in induction of *P_AOX1_* [100]. Although these two studies obtained lower gene expression levels than for methanol-induced *P_AOX1_*, the goal here resides in the development of a methanol-free bioprocess that offers a safer alternative, hugely relevant to industry.

Yu et al. [101] reported the separate effects of the co-overexpression of nine proteins under *P_AOX1_* regulation, on *P_GAP_*-driven k-carrageenase production, including seven chaperones (Pdi: protein disulphide isomerase; Ire1: endoplasmic reticulum stress transducer; ero1: endoplasmic reticulum oxidoreductase; Kar2: immunoglobulin-binding protein; Aha1: activator of Hsp90 ATPase; Ypt6; GTPase; Prx1: thioredoxin-linked peroxidase) and two transcription factors Yap1 and Rpn4 (proteasome subunit transcription factors) [101,102]. Overexpression of Rpn4 yielded a 1.36-fold (7.07 U/mL) increase in the production of active k-carrageenase in the medium, and Yap1 yielded a 1.72-fold increase (7.42 U/mL), in contrast with the 2.73 U/mL obtained without methanol induction of auxiliary genes (transcription factors or chaperones). The cell engineering approach used in this work is noteworthy given the ability to compare the effect of co-expression of auxiliary genes in the same strain, using the methanol-inducible promoter *P_AOX1_* for these and a constitutively expressed promoter such as *P_GAP_* for the rProt of interest.

Consequently, the modulation of expression of genes encoding transcriptional regulators offers a variety of alternatives enabling an increment in the rProt production yield. However, the high number of genes affected by the modulated expression of these factors should be considered [76,79,103]. Given that, in general, the comparisons are performed under optimised process conditions for the wild-type strain, the results obtained by adapting the bioprocess to other transcriptional variants of *K. phaffii* are unknown [23].

## 4. Improving Protein Folding and Secretion

The main hindrances in the production and secretion of proteins are the folding and processing of complex proteins in the ER. When proteins fold inappropriately or exceed the capacity of the ER, unfolded proteins can accumulate and tend to form aggregates. The cellular response generally involves synthesis and induction of folding-assisting proteins such as chaperones or foldases [13,104,105]. To overcome this bottleneck, different researchers have proposed the modification of some genetic factors such as codon sequence optimisation, promoter selection [106], gene dosage [107], and co-expression of folding helper proteins to improve rProt expression [108]. Some examples of these approaches are summarised in Table 3.

Regarding codon sequence optimisation, the codon usage bias can be adapted to the host [106], and this strategy is widely used with the assistance of software tools focused on replacing rare codons with frequently used codons in *K. phaffii* [109]. Karaoğlan and Erden-Karaoğlan [106] described a model for the expression of the protein endo-poly-galacturonase (Pgl) of *A. niger*, whose sequence was subjected to codon optimisation, evaluating its performance under the regulation of two promoters (*P_AOX_* and *P_ADH2_*). The highest production level was achieved with the codon-optimised *PGL* using the *pADH2* obtaining a productivity of 42.33 U/mL (4-fold increase) in shake flasks.

The co-expression of chaperones can promote the correct folding of rProts and reduce the intracellular aggregation of proteins [101]. The protein disulphide isomerase (Pdi1) present in the ER plays a crucial role in restoration and isomerisation of disulphide bonds in nascent proteins [7,101]. Lan et al. [110] showed that the expression of the marine *Streptomyces* sp. lipase Mas1 increased 1.7-fold when it was co-expressed with the chaperone Pdi1, compared to the control strain harbouring only *MAS1* [111].

Another chaperone protein frequently co-expressed with rProt is Kar2, a homologue of the mammalian immunoglobulin-binding protein (BiP) [108,111] that acts as a sensor for misfolded proteins, participating in protein folding or directing recalcitrant proteins to the ER-associated degradation pathway (ERAD) [112]. An example of co-expression was reported by Sellada et al., expressing a hydrophobin class II (Hfbi) which, when co-expressed with Kar2, resulted in a 22-fold increase in productivity with respect to the strain without the chaperone [108].

Another strategy that also improves protein folding and secretion is the expression of transcription factors such as Yap1 and Hac1, as it was presented in the previous section. However, when these are expressed together with other folding facilitator proteins such as Pdi1 and Kar2, although it was expected to result in an increase in the production of the rProt of interest, the work of Sun et al. [81] and Duan et al. [113] showed that the rProt productivity was either maintained or decreased.

For this reason, many researchers have implemented more than one modification simultaneously, integrating the co-expression of chaperones and/or foldases, in conjunction with other genetic manipulation tools such as optimisation of codon usage, gene copy number, co-expression/modulation of transcription factors, and variation in culture conditions with the purpose of improving rProt productivity [108,111,114,115]. A clear summary example is reported by Ben Azoun et al. [116], with the expression of the rabies virus glycoprotein (RABV-G), where the molecular factors addressed were gene optimisation, secretion signal sequence, gene copy number, and the co-expression of different proteins. Gene optimisation increased productivity at approximately 2.1-fold, and the overexpression of the two folding factors *PDI1* or *ERO1* remarkably increased the expression level of RABV-G by 9.5-fold and 3.3-fold, respectively, in the high copy strains of RABV-G (more information available in Table 3).

These findings demonstrate that there is no combinatorial strategy that provides equal benefit for the secretion of all recombinant proteins, and further research is necessary to find the most suitable strategy to obtain the desired recombinant product.

**Table 3:** Improving heterologous protein production by protein folding and secretion.

Auxiliary Gene	Modification	Pathways Involved	Heterologous Product	Production (Fold Change)	Operation Mode	Scale	Strain	Ref.
*PDI1*	OE	Folding	Fab (S)	+1.9	Batch	Flask	X-33	[117]
*PDI1* *PDI1* w/ CN	OE	Folding	Na-ASP1	+3.2 + 7.9	Batch	Flask	X-33	[118]
*KAR2* *PDI1* *PDI1/KAR2*	OE	Folding	A33scFv (S)	+3 No effect No effect	Batch	2.5 L Bioreactor	GS200	[119]
*PDI1* *KAR2* *ERO1* *SEC1* *SLY1*	OE	Folding and Trafficking	IL2-HSA (S)	+2.2 +1.9 +2.3 +2.5 +1.9	Batch	Flask	GS115	[111]
*PDI1* + *CN* *KAR2* + *CN* *ERO1* CN	OE	Folding	Hydrophobin (S)	+7.8 +22 +30 No effect	Batch	Flask	GS115	[108]
*PDI1* *ERO1* CN	OE	Folding	hLYZ (S)	+2.43 +2.30 +1.57	Batch	5 L Bioreactor	GS115	[7]
*YDJ1* *SSA1* *SEC63* *KAR2*	OE	Folding and Trafficking	CalB (S)	+1.6 +1.4 +1.4 −0.7	Batch	Flask	GS115	[115]
*PDI1* *ERO1* *GPX1* *GLR1* *YAP1*	OE	Folding	RABV-G (S)	+9.5 +3.3 +8.2 +1.2 No effect	Batch	Flask	KM71H/GS115	[114,116]

Abbreviations: *PDI1*: protein disulphide isomerase; *KAR2*: immunoglobulin-binding protein; *ERO1*: endoplasmic reticulum oxidoreductase; *SEC1*: Sm-like protein involved in docking and fusion of exocytic vesicles; *SLY1*: hydrophilic protein involved in ER/Golgi vesicles trafficking; *GPX1*: glutathione peroxidase; *GLR1*: glutathione reductase; *YDJ1*: type I HSP40 co-chaperone; *SSA1*: Hsp70 family ATPase involved in protein folding; *SEC63*: protein-transporting protein; Fab: antibody fragment; Na-ASP1: *Necator americanus* secretory protein; A33scFv: A33 single-chain antibody fragment; IL2-HSA: human albumin fusion protein; hLYZ: human lysozyme; CalB: *Candida antarctica* lipase B; RABV-G: rabies virus glycoprotein; CN: gen copy number.

## 5. Conclusions

*K. phaffii* has reached a well-recognised position as a successful platform to produce biotechnologically and commercially attractive products (as shown in Table 1, Table 2 and Table 3), mainly due to its ability to produce a diversity of functionally active heterologous proteins, ranging from proteins of microbial origin to complex eukaryotic proteins with several applications [3,120,121].

Metabolic modifications aided by gene codon optimisation, promoter engineering, and genomic engineering with different synthetic biology tools have made it possible to implement new metabolic routes with the objective of developing more robust strains, with improved capabilities and fitness, which can provide higher rProt productivity levels and mitigate issues related to methanol metabolism, the catabolism of cheap raw carbon sources (e.g., xylose, cellulose), and also the potential conversion of *K. phaffii* into an autotrophic microorganism [64].

Further study of metabolic modifications directed to assimilation routes of carbon and energy sources in response to the culture media will have a repressive or derepressive response to the highly used *P_AOX1/2_* induction system and to the metabolic system in general.

On the other hand, modifications to increase/improve protein folding and secretion, such as codon usage optimisation, increase in gene copy number, co-expression/modulation of transcription factors, co-expression of chaperones, and modification of culture conditions have allowed the expression of a wide range of proteins with diverse application fields. However, the use of one or many of these modifications does not ensure an increase in rProt expression, especially in the case of proteins that are difficult to express, such as antibodies, membrane proteins, toxins, and proteins with non-standard amino acids, among others [107,109,114,116]. Each protein appears to be as unique as the combinatorial strategies that can be incorporated to enhance its expression. So far, there is no combinatorial strategy that enhances all recombinant proteins equally, and further research is needed in order to find the right strategy for the specific desired product, turning the optimisation into a product-based approach [13].

Furthermore, due to the variety of available strains of *K. phaffii* used to produce recombinant proteins, it is necessary to have a model that integrates information related to cell behaviour in a way that is capable of developing hypotheses focused on optimisation of production processes [122]. The genome-wide metabolic model characterises cell physiology, which allows obtaining valuable information on metabolism and designing possible strategies to improve a strain through in silico simulations [123]. This computational approach, combined with synthetic biology techniques, potentially forms a basis for the rational analysis and design of the *K. phaffii* metabolic network to improve recombinant protein production [124,125].

Finally, the latest advances and improvements addressed in this review have been reported mainly at laboratory scale (flask) with only a few examples at bioreactor level or high cell density. This highlights the need to expand the research scope on the scale-up of these processes, in order to determine cultivation parameters suited for higher-scale and for industrially relevant cultivation modalities (fed-batch and, increasingly, continuous mode) and strain performance in controlled conditions. In this regard, engineering tools such as response surface methodology can be used, as they have been successfully applied to improve *K.-phaffii*-based bioprocesses [126,127,128,129].

## Figures and Tables

**Table 1:** Improving heterologous protein production by modifying metabolism and energy supply.

Auxiliary Gene	Modification	Pathways Involved	Heterologous Product	Production (Fold Change)	Operation Mode	Scale	Strain	Ref.
*A. Engineering of the MUT pathway*								
*P_AOX_DAS*	OE	MUT	HRP (S)	+0.94	Batch	5 L Bioreactor	CBS7435 (MUTs)	[37]
			CalB (S)	+0.56				
*P_AOX_FLD*	OE	MUT	HRP (S)	+1.21				
			CalB (S)	+1.07				
*DAS1*, *DAS2* *DAS1/2*	Del	MUT	eGFP	+1.3 +1.2 +1.15	Batch	Flask	CBS7435 (MUTs)	[38]
*P_FLD1_ADH2*	OE	MUT	cFab-vHH (S)	+2.69	Fed-batch	1 L Bioreactor	CBS2612 (MUT^−^)	[39]
*FDH*	Del	MUT	β-galactosidase	+2	Batch	Flask	GS115 (MUT^+^)	[40]
*B. Engineering of co-substrate catabolic pathways*								
*ATG30*	Del	MUT	Xylanase (S)	+3	Batch	Flask	GS115 (MUT^+^)	[41]
*C. Engineering of co-factor metabolism*								
*P_GAP_POS5*	OE	PPP	Fab (S)	+2	Chemostat	2 L Bioreactor	X-33 (MUT^+^)	[27]
*P_GAP_ZWF1-P_GAP_ SOL3*	OE	PPP	hSOD	+3.8	Batch	Flask	SMD1168H	[42]
*P_AOX_SOL3-P_AOX_GND2*	OE	PPP	hIFN-γ (S)	+2.2	Fed- batch	1 L Bioreactor	GS115 (MUT^+^)	[43]
*D. Synthetic biology for alternative carbon source catabolism*								
*P_GAP_XI*	Ex	Xylose path.	β-mannanase (S)	+1.36	Batch	Flask	GS115 (MUT^+^)	[44]

Abbreviations: HRP: horseradish peroxidase; CalB: *Candida antarctica* lipase B; eGFP: enhanced green fluorescent protein; *DAS1*: dihydroxyacetone synthase 1; *DAS2*: dihydroxyacetone synthase 2; *FLD:* formaldehyde dehydrogenase; cFab-vHH: camelid antibody fragment; *ADH:* alcohol dehydrogenase; *FDH*: formate dehydrogenase; *POS5*: NADH kinase; *ZWF1*: glucose-6-phosphate dehydrogenase; *SOL3*: 6-gluconolactonase; *GND2*: 6-phosphogluconate dehydrogenase; *XI*: xylose isomerase; MUT: methanol utilisation pathway; PPP: pentose phosphate pathway; hSOD: cytosolic human superoxide dismutase; IFN-γ: recombinant human interferon gamma; (S) secreted protein; OE: gene overexpression; Del: gene deletion; Ex: expression by insertion.

**Table 2:** Improving heterologous protein production by transcription factor modification.

Auxiliary Gene	Modification	Pathways Involved	Heterologous Product	Production (Fold Change)	Operation Mode	Scale	Strain	Ref.
*P_SUT2_-MXR1*	OE	MUT and sterol biosynthesis	eGFP	+1.18	Batch	Flask	GS115	[75]
*FHL1p*	OE	Ribosome biosynthesis, processing	Phytase (S) Pectinase (S) mRFP	+1.2 +1.35 +1.31	Batch	Flask	GS115	[76]
*P_AOX2_*-*MXR1*	OE	MUT	scFv	+2.7	Batch	Flask	KM71	[77]
*ATT1*	Del	Cellular fitness modulation	IgG1 (anti-HER2)	+1.5	Fed-batch	15 L Bioreactor	Gly. Eng.	[78]
*YAP1*	OE	Oxidative stress response	Trypsinogen (S)	+2.0	Batch	Flask	X-33	[79]
*AFT1*	OE	Secretion and carbohydrate metabolism	Carboxylesterase (S)	+2.5	Fed-batch	1 L Bioreactor	CBS7435	[80]
*HAC1*	OE	UPR	Bovine lactoferrin (S)	+5.0	Fed-batch	5 L Bioreactor	GS115	[81]
*HAC1*	OE	UPR	Thrombomodulin Adenosine A2A mIL-10 (S) Trans-sialidase (S)	+1.9 +1.18 +2.2 +2.1	Batch	Flask	GS115	[82]
*HAC1*	OE	UPR	HsCstp HsCtr1p OsCstp	+2.1 +1.7 +1.5	Fed-batch	1 L Bioreactor	CBS7435	[83]
*HAC1*	OE	UPR	Lysozyme (HYL) (S)	+2.13	Batch	Flask	KM71H	[84]
*HAC1*	OE	UPR	Lactone esterase (S)	+1.8	Batch	Flask	NRRL-Y-11430	[85]
*HAC1*	OE	UPR	elastase (S)	+1.8	Batch	Flask	GS115	[86]

Abbreviations: MUT: methanol utilisation pathway; UPR: unfolded protein response pathway; Gly. Eng.: glycoengineered strain; *P_SUT2_*: sterol uptake protein 2 promoter; *MXR1*: methanol expression regulator 1; eGFP: enhanced green fluorescent protein; *FHL1p*: regulator of ribosomal protein transcription; mRFP: monomeric red fluorescent protein; scFv: single-chain variable fragment; *ATT1*: GAL4-like transcriptional regulator; IgG1 (anti-HER2): IgG1 monoclonal antibody that targets the HER2 receptor; *YAP1*: oxidative stress response transcription factor; *AFT1*: activator of ferrous transport transcription factor; *HAC1*: UPR-regulating transcription factor; mIL-10: mouse interleukin-10; HsCstp: human CMP-sialic acid transporter; HsCtr1p: human copper transporter Ctr1; OsCstp: rice CMP-sialic acid transporter; (S) secreted protein.

## Data Availability

Not applicable.
